# Antiproliferative Effect of *Phellodendron amurense* Rupr. Based on Angiogenesis

**DOI:** 10.3390/life12050767

**Published:** 2022-05-21

**Authors:** Ľudmila Balážová, Slavomír Kurhajec, Martin Kello, Zdenka Bedlovičová, Martina Zigová, Eva Petrovová, Katarína Beňová, Ján Mojžiš, Jarmila Eftimová

**Affiliations:** 1 Department of Pharmaceutical Technology, Pharmacognosy and Botany, University of Veterinary Medicine and Pharmacy in Košice, 041 81 Košice, Slovakia; slavomir.kurhajec@uvlf.sk (S.K.); jarmila.eftimova@uvlf.sk (J.E.); 2 Department of Pharmacology, Faculty of Medicine, P. J. Šafárik University, Trieda SNP 1, 040 11 Košice, Slovakia; martin.kello@upjs.sk (M.K.); martina.zigova@upjs.sk (M.Z.); jan.mojzis@upjs.sk (J.M.); 3 Department of Chemistry, Biochemistry and Biophysics, University of Veterinary Medicine and Pharmacy in Košice, 041 81 Košice, Slovakia; zdenka.bedlovicova@uvlf.sk; 4 Department of Morphological Disciplines, University of Veterinary Medicine and Pharmacy in Košice, 041 81 Košice, Slovakia; eva.petrovova@uvlf.sk; 5 Department of Biology and Physiology, University of Veterinary Medicine and Pharmacy in Košice, 041 81 Košice, Slovakia; katarina.benova@uvlf.sk

**Keywords:** angiogenesis, berberine, cancer, *Phellodendron amurense*, CAM

## Abstract

*Phellodendron amurense* Rupr. is medicinal plant used for supplemental therapy of various diseases based on their positive biological activities. The aim of this study was evaluated the main metabolite, safety of application and anticancer potential. Berberine was determined by HPLC as main alkaloid. Harmful character was determined by irritation test in ovo. The potential cancerogenic effect was studied in vitro on a cellular level, in ovo by CAM assay and in vivo on whole organism *Artemia franciscana*. Extract from the bark of *Phellodendron amurense* showed antiproliferative and antiangiogenic effects. The results of our work showed promising anticancer effects based also on the inhibition of angiogenesis with minimum negative effects.

## 1. Introduction

The plant *Phellodendron amurense*, Rupr. (PA) from the family Rutaceae is one of the major plants in traditional Chinese medicine. So-called huang-bo or huang-bai have been used traditionally in folk medicine for hundreds of years as anti-inflammatory, spasmolytic and antidiarrheal agents; as a bitter tonic for stomach problems; and in the treatment of dyspepsia, hepatitis, dysentery, gynaecological inflammation, meningitis, gastreoenteritidis, tuberculosis or pneumonia [1,2].

**Figure 1:**
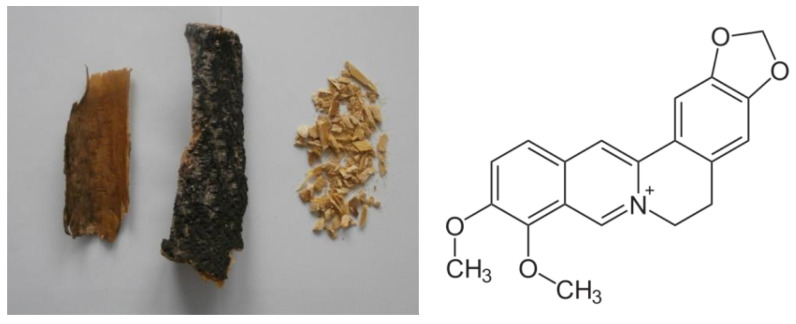
Bark of *Phellodendron amurense* Rupr and alkaloid berberine.

PA bark (Figure 1) contains mainly isoquinoline alkaloids such as phellodendrine, berberine (Figure 1), oxyberberine, epiberberine, jatrorrhizine, magnoflorin and palmatine [2,3]. This plant is also the source of limonoids (limonin, kihadanin, obacunone [4]), isovanillin, ferulic acid, ethyl caffeate and other phenolic substances [5,6]. These active compounds possess many different biological activities, but they exhibited low toxicity to normal cells and tissues [7].

The *Phellodendron amurense* bark extract is also a potential therapeutic agent to protect cartilage against the osteoarthritic progression [1,8,9]. There are a few preparations with extract from bark of *Phellodendron amurense* Rupr. used for the anti-inflammatory and analgesic properties available on the market (e.g., Nexrutine^®^, Relora^®^) [10]. It was shown, that total alkaloids obtained in cortex had protective effect on stomach ulcer compared to commercially used omeprazole [11]. The aqueous extract of *Cortex phellodendri* and its main alkaloid berberine are important immunomodulators due to their induction and stimulation of the antiviral state [12]. Several studies have demonstrated their anticancer property on prostate cancer [13,14], pancreatic cancer [15,16], breast cancer [6] and skin cancer [2,17]. It was observed that PA inhibits the proliferation of lung and prostate cancer cells by various mechanisms, including the inhibition of cell migration and invasion, induction of apoptosis and cell cycle arrest via a regulation of multiple pathways, such as changes in reactive oxygen species generation, matrix metalloproteinase regulation, p53 activation, NF-kB signal activation and DNA or RNA binding [18,19]. Although the mechanism is not fully understood, some of the activity against cancer cells is beginning to be deciphered [19].

In the initial stage of the carcinogenic process, the nutrition of a tumor tissue depends on the diffusion from the surrounding host tissues. Later, the formation of the vascular system is existentially important for tumor growth. From that time, the research community made significant progress in studying the relationship between angiogenesis and cancer. The number of published papers dealing with cancer and angiogenesis has rising dramatically, which is proof that this topic is constantly of significant interest. Angiogenesis is essential for the progression of tumor growth and metastasis, too. In contrast to normal vascular beds, the new tumor vasculature provides an inefficient blood supply to the tumor, characterized by a highly disorganized structure, increased permeability, abnormal spacing, decreased and abnormal pericyte–endothelial cell adhesions, irregular basement membrane structures and the incorporation of bone-marrow-derived endothelial progenitor cells into the new microvasculature [20]. The anti-angiogenic therapy can be potentially used for all types of cancer because the tumors need the formation of vasculature in order to grow [21].

The avian embryos play key role in basic medicinal research. This alternative animal model is cost-effective and easily available, which may be used in the area of testing various materials, for example, plant extracts [22]. The chick chorioallantoic membrane CAM is a simple and rich vascularized extra-embryonic membrane which surrounds the developing chick embryo [23]. This Hen’s Egg Test (HET-CAM test) was first reported by Luepke [24]. It was developed as a rapid, sensitive and inexpensive test the irritancy potential of substances [25]. The CAM model has been accepted instead of Draize test on rabbits with respect to the 3Rs (Replacement, Reduction, Refinement) [26]. Chick embryo development takes place outside the mother’s body, and therefore the effect of a substance or biomaterial is observed directly on the embryo without the influence of or detrimental effect on the mother’s metabolism [27]. The CAM is used for a wider extent of research, including assessing the molecules regulating the formation of blood vessels for use in the treatment of tumors, drugs delivery, angiogenic and anti-angiogenic molecules [28].

The genus *Artemia* from the family Artemiidae is widely used as a model organism for biochemical, physiological, genetic and ecological studies. This genus is able to adapt to wide range of salinity (5–250 g/L) and temperatures (6–35 °C) [29]. *Artemia franciscana* is suitable for monitoring toxicity because it is very sensitive to many chemical substances [30].

The aim of our research was to determine the concentration the secondary metabolites presented in *Phellodendron amurense* Rupr. and tested their harmless character and its biological activities, such as its anti-angiogenic and antiproliferative potential. To our knowledge, this is the first time the potential inhibition of the formation of blood vessels by *Phellodendron amurense* is being reported. Our results could contribute to the development of new drugs based on these plant extracts.

## 2. Materials and Methods

### 2.1. Preparation of Basic Extracts

The dried trunk bark of *Phellodendron amurense* Rupr. used in the experiments was obtained from Hlorofitum company, Ukraine. The PA bark was milled for 1 min at 25,000 rpm by electric grinder (Sencor SCG 1050WH, Yiwu, China), and the achieved powder was subsequently sieved by stainless-steel sieve with an aperture of 1000 μm. From milled PA bark, we prepared four extracts according to the principles of pharmacy practice. *Decoctum*: boiling PA bark (20 g) in water (100 mL) for 30 min; *Infusum*: boiling PA bark (20 g) in water (100 mL) for 5 min and then to macerate for 45 min at room temperature; *Infusum frigidum*: maceration PA bark (20 g) in water (100 mL) for 30 min at room temperature; *Tinctura*: maceration PA bark (20 g) in 60% ethanol (SLAV000003, Labo, Bratislava, Slovakia) (100 mL) for 7 days.

### 2.2. Determination of Isoquinoline Alkaloids

We mixed 40 mL of the *Tinctura* with 1 mL of 26% ammonia (SLAV000036, Labo, Bratislava, Slovakia). This solution was shaken separately three times with 15 mL of chloroform (SLAV000027, Labo, Bratislava, Slovakia). The combined chloroform fractions were dried at temperature not exceeding 50 °C by rotary evaporator (Heidolph, Schwabach, Germany). The dried residue was subsequently dissolved in 50 mL of methanol (SLAV000071, Labo, Bratislava, Slovakia). The absorbance of this solution was measured by spectrophotometer at 348 nm (Beckman DU 530 UV/VIS, Delta Electronics, Pudong Shanghai, China). The concentration of isoquinoline alkaloids in the basic extracts was expressed as berberine (berberine hydrochloride, B3251, Sigma Aldrich, Darmstadt, Germany) by using a calibration graph. The calibration graph (linear correlation) was made by measuring the absorbance of standard berberine solutions in methanol of different concentrations (1 × 10^−3^ M, 1 × 10^−4^ M, 5 × 10^−4^ M, 1 × 10^−5^ M, 2.5 × 10^−5^ M) at 348 nm [31].

### 2.3. UHPLC Analysis

#### 2.3.1. Extraction Procedure for UHPLC Analysis

The tincture was filtered through the 0.22 μm PTFE membrane filter, before the use in the analysis.

#### 2.3.2. Quantitative Analysis

For analysis, the UHPLC Dionex UltiMate 3000 system with DAD detector at a wavelength of 346 nm (Thermo Scientific, Rockford, IL, USA) was used. For separations, the reverse-phase column YMC Meteoric Core C18 Bio (150 × 4.6 mm) with a particle size of 2.7 μm was utilized. The injection volumes were 5 μL. The specificity of the method was studied using standard samples of berberine (purchased from Sigma Aldrich, Germany, B3251). In the range of concentrations between 1.0 and 0.025 mg/mL. For the mobile phase, water (eluent A) and acetonitrile (eluent B) (A/0626/17, Fisher Scientific, Loughborough, Leics, United Kingdom) was used, using gradient elution. In the time of 0–2 min, 80% of eluent A was used with the flow rate range of 1 to 0.1 mL/min, then 50% of eluent A was used using the flow from 0.1 to 1 mL/min. The repeatability of sample measurements was carried out using three replicates of the same sample and was expressed as percentage of relative standard deviation (%RSD). The quantity of berberine was calculated by comparing their peak areas with those of standard solutions.

### 2.4. Determination of Polyphenols

The concentration of total polyphenols in the basic extracts of PA bark (*decoctum*, *infusum*, *infusum frigidum*, *tinctura*) was measured by Singleton method using Folin–Ciocalteu reagent (1.09001, Sigma Aldrich, Darmstadt, Germany) by spectrophotometer (Beckman DU 530 UV/VIS, Delta Electronics, Pudong Shanghai, China) at 760 nm and calculated as concentration of hyperoside [32].

### 2.5. MTS Assay

The effect of *Phellodendron amurense* bark tincture on cell proliferation was determined using the MTS [3-(4,5-dimethylthiazol-2-yl)-5-(3-carboxymethoxyphenyl)-2-(4-sulfophenyl)-2H tetrazolium)] test. Cells (HeLa, HCT, MCF-7, A549 and 3T3) were seeded in 96-well plates at a density of 5 × 10^3^ cells/well. Then, 24 h after cell seeding, the different concentrations (10–500 µg/mL) of tincture were added, and the plates incubated at 37 °C for an additional 72 h. Vehicle (60% ethanol) final concentration range was tested as internal control. At the end of the treatment period, MTS reagent (Promega, Madison, WI, USA) was added to each well, and the plates were incubated at 37 °C for 4 h in 5% CO_2_. Cell proliferation was evaluated by measuring the absorbance at 490 nm using an automated CytationTM 3 cell imaging multi-mode reader (Biotek, Winooski, VT, USA). A nonlinear regression method was used to calculate IC50 values (concentrations required to produce 50% growth inhibition).

### 2.6. Cell Proliferation Analysis

To analyse the proliferation activity of HeLa, HCT116, MCF-7, A549 and 3T3 cells, the CellTrace^TM^ Yellow Cell Proliferation Kit for flow cytometry was used (Thermo Scientific, Rockford, IL, USA). The cells (1 × 10^6^) were resuspended in 1 mL staining solution with final concentration 10 µM and incubated for 20 min at 37 °C in the dark. After incubation, cells were washed in a complete culture medium and incubated again for 5 min at 37 °C. After second incubation, supernatant was removed by centrifugation, and the pellet was resuspended in fresh, pre-warmed complete culture medium and seeded in 6-well plates for 24 h. Afterwards, seeded cancer cells were treated with PA tincture (IC50) and murine 3T3 fibroblasts with the highest IC50 from cancer lines and analysed three different times (24, 48 and 72 h). Cells were also treated with vehicle (60% ethanol) in same *v*/*v* %. For analysis of stained cells, a BD FACSCalibur flow cytometer (BD Biosciences, San Jose, CA, USA) was used. A minimum of 1 × 10^4^ events were analysed per analysis and all experiments were performed in triplicates.

### 2.7. Cell Cycle Analysis

For the cell cycle analyses, HeLa, HCT116, MCF-7, A549 and 3T3 cells were harvested in three different time-points (24, 48 or 72 h) after PA tincture treatment (IC50) or vehicle, washed in cold PBS and fixed in cold ethanol (70%). Afterwards, samples were stored at −20 °C until analysis (minimal 24 h). Prior to each analysis, cells were stained with solution containing 0.5 mg/mL ribonuclease A, 0.2% final concentration Triton X-100 and propidium iodide 0.025 mg/mL in 500 µL PBS (Merck, Darmstadt, Germany) and incubated for 30 min at room temperature in the dark. The BD FACSCalibur flow cytometer (BD Biosciences, San Jose, CA, USA) was used to analyse stained cells. A minimum of 1 × 10^4^ events were analysed, and all experiments were performed in triplicates.

### 2.8. Artemia Franciscana Test

Screening *Artemia franciscana* test was used to determine cytotoxicity of PA. We extracted 1 g of PA bark in 100 mL of 100 °C seawater (NaCl 23.900 g/L, MgCl_2_·6H_2_O 10.830 g/L, CaCl_2_·6H_2_O 2.250 g/L, KCl 0.680 g/L, Na_2_SO_4_·10H_2_O 9.060 g/L, NaHCO_3_ 0.200 g/L, SrCl_2_·6H_2_O 0.040 g/L, KBr 0.099 g/L, H_3_BO_3_ 0.027 g/L; Ph-8.31) for 10 min to prepare extract. We filled the Petri dishes (diameter 60 mm) with 10 mL of extracts and seawater (as control), and we put the 10 nauplii of the brine shrimp into each of them. In each tested group, 50 nauplii were used divided into the 5 Petri dishes. Petri dishes with nauplii in the tested extract or seawater were put into the thermostat with temperature 20 ± 1 °C. We observed the lethality of *A. franciscana* in tested extracts at 24, 48, 72 and 96 h [33].

### 2.9. Preparation of Extracts for CAM Assays

The prepared tincture of PA bark was evaporated by a rotary evaporator (Heidolph, Schwabach, Germany) at 50 °C. After the evaporation of solvent, the dry extract of PA was diluted in saline solution (SLAV000004, Labo, Bratislava, Slovakia) to the final concentrations of 0.1, 1.0 and 10.0 mg/mL.

### 2.10. Preparation of Eggs

Freshly laid fertilized white Leghorn chicken eggs (120 pcs) were purchased from the local hatchery (Parovske Haje, Nitra, Slovakia). The entire exterior of the eggs was cleaned and disinfected by ethanol (70%). They were incubated in a blunt end position in forced-draft incubator (River ET549/A, River Systems, Italy) with automatic continuous rocking mechanisms (rotating of the eggs every 3 h) and with environmental standard conditions of 37.5 °C temperature and 60% relative humidity. On the next day, 2 mL of albumen was removed through the eggshell by a syringe with a needle (20 G) from each egg. Subsequently, the hole in the shell was covered with paraffin. The eggs were returned to incubator with same condition as written above and with daily monitoring to discard dead embryos. On the fourth day of the incubation period, the small part of eggshells from the blunt end of each egg were removed by special scissors, and the inner shell membranes were carefully removed by forceps without destroying vascular chorioallantoic membrane CAM. Windowed CAM assay was performed. On the embryonic day 8, CAM was exposed to working solution and saline solution as control. After 72 h, the changes in CAM were photographed by stereomicroscope Olympus SZ61 (Olympus corporation, Tokyo, Japan), digital camera ARTCAM-300MI (ARTRAY, Tokyo, Japan) and software Quick Photo 2.3 (PROMICRA, Prague, Czech Republic). Then, the chick embryos were humanely killed by decapitation with scissors disposed of safely. The chick embryo is considered as experimental model, as they are exempt from the horizontal legislation on the protection of animals used for scientific purposes (2010/63/EU), as well as applicable laws in the United States, it means that no animal protocol approval for the chick embryo is requested.

### 2.11. Vasoactivity

The test extracts 0.1, 1.0 and 10.0 mg/mL of PA in saline solution were dropped gently onto the CAM vessels in a volume of 20 µL on the embryonic day 8. Saline solution (20 µL) was used as control. After the application of the test extract, the blood vessels and capillary system of CAM were photographed a stereomicroscope Olympus SZ61 (Olympus corporation, Tokyo, Japan), digital camera ARTCAM-300MI (ARTRAY, Tokyo, Japan) and software Quick Photo 2.3 (PROMICRA, Prague, Czech Republic) after 0.5, 2 and 5 min from application. To minimalized subjective observations, two independent scientists evaluated irritant effects (totally 5 eggs per solutions). As described by Luepke (1985), the numerical time-dependent scores for irritant effect including hyperaemia, haemorrhage and coagulation were summed to give a single numerical value which indicates the irritation assessment [24].

### 2.12. Angiogenesis

The PA dissolved in isotonic saline solution (1 mg/mL) was dropped (20 µL) into the ring on the CAM surface. Ten eggs were used for PA extract and control (20 µL saline solution). The holes in the eggshells were immediately covered using an electrical insulation tape and the eggs were returned to the incubator with the same humidity and temperature settings. After 72 h, the blood vessels were observed and photographed to measure the anti-angiogenic effect. The photographs were obtained using a stereomicroscope Olympus SZ61 (Olympus corporation, Tokyo, Japan), digital camera ARTCAM-300MI (ARTRAY, Tokyo, Japan) and software Quick Photo 2.3 (PROMICRA, Prague, Czech Republic). For calculation of anti-angiogenic effect, ImageJ 1.48 software was used (NIH Image, Bethesda, MD, USA). The effect on angiogenesis was evaluated by two methods to minimalize the subjectivity percentage of avascular zone and vessel sprouting. The avascular zone (area without blood vessels) of the chorioallantoic membrane was evaluated by assessing the pixels above a specific threshold [34]. The percentage inhibition of avascular zone was calculated using the following Formula (1):

(1) $$\%\;inhibition\;of\;vascularization=\frac{\%\;\mathrm{vascularized}\;\mathrm{area}\;\mathrm{of}\;\mathrm{treated}\;\mathrm{sample}}{\%\;\mathrm{vascularized}\;\mathrm{area}\;\mathrm{of}\;\mathrm{control}\;\mathrm{sample}}\times 100$$

Vessels’ sprouting–branching frequency was counted as the number of microvessel branch points in every sample after three days of exposure. Fold induction of vessel sprouting was determined as the ratio between the number of vessels sprouting in sample after incubation to the number of vessels sprouting in the same sample before application of the sample. The enumeration of the vascular area and vessel branching was recorded blindly by two independent observers to minimize subjective mistakes.

### 2.13. Statistical Analysis

The average score and SEM were calculated and graphically interpreted in graphs. For all the measurements, one-way ANOVA followed by a Dunnet´s post hoc test was used to assess the statistical significance of the difference between each group. A statistically significant difference was considered at the level of *p* < 0.05 (*), *p* < 0.01 (**) and *p* < 0.001 (***) vs. untreated control cells and *p* < 0.05 (+) and *p* < 0.01 (++) vs. vehicle-treated cells. Software GraphPad Prism 5 (GraphPad Software, San Diego, CA, USA) was used for statistical analysis.

## 3. Results

### 3.1. Phytochemical Analysis

The *Phellodendron amurense* bark extract was prepared by different procedures in order to find the most favourable concentration of substances responsible for antiproliferative and antiangiogenic effects. The concentration of total polyphenols and alkaloids differed according the type of extraction procedure. The sequence of extracts from the lowest to the highest content of polyphenols was as follows: *tinctura* 0.50 ± 0.012 g/L, *infusum frigidum* 0.70 ± 0.017 g/L, *infusum* 1.37 ± 0.033 g/L and *decoctum* 1.61 ± 0.039 g/L. In case of isoquinoline alkaloids, the highest concentration was observed in ethanolic *tincture* 49.4 ± 0.75 mg/L and the lowest in *infusum* 2.74 ± 0.05 mg/L (Table 1).

The content of polyphenols is not in correlation with the concentration of isoquinoline alkaloids in different extracts (R^2^ = 0.3804). According to these results, the highest amount of isoquinoline alkaloids is in the tincture. Therefore, only alcohol extracts were used to determine the concentration of berberine by HPLC, as well as for further in vitro and in ovo methods.

**Figure 2:**
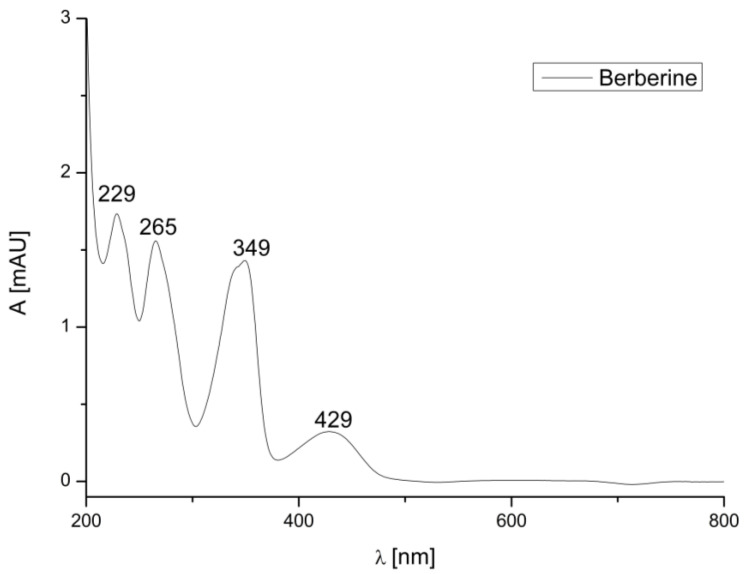
UV/Vis spectra of berberine.

**Figure 3:**
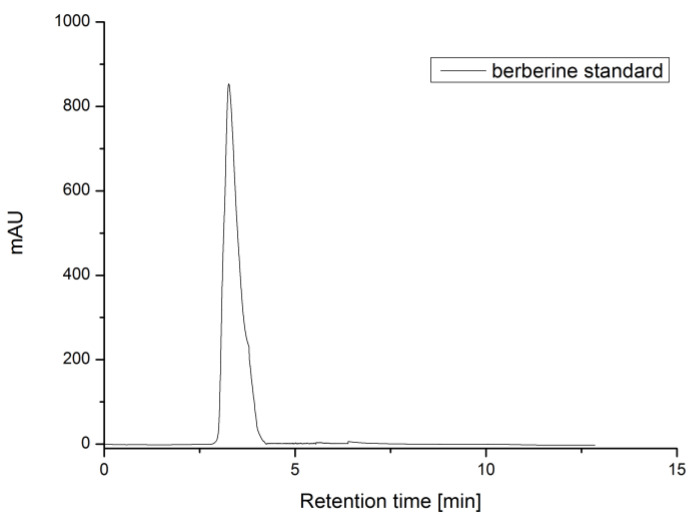
Berberine standard chromatogram.

**Figure 4:**
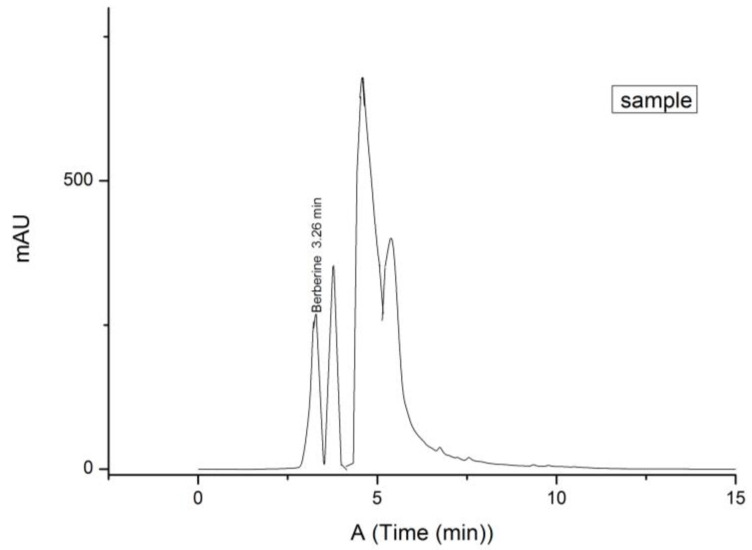
Chromatogram of ethanolic tincture of *Phellodendron amurense* bark.

To determine the retention time and peak area, three repeated analyses of standards were carried out under optimal HPLC conditions (Figure 2, Figure 3 and Figure 4). The calibration curve of berberine was linear in a relatively wide concentration range (0.025–1.000 mg/mL), the linear regression was had a high correlation (R^2^ = 0.9978) and %RSD was 2.97. LOD and LOQ were defined as the signal-to-noise ratios (S/Ns) of 3 and 10, respectively. According to the calculation, the LOD and LOQ values of berberine were 0.00556 and 0.01684 mg, respectively.

The berberine content of the PA tincture was determined using the calibration curve of standard solutions with known concentrations. The content is expressed as an average value of three analyses ± SD in mg of berberine per kg of dry weight (mg/kg DW), and the values were 32.96 mg/kg DW ± 0.2 and is 28.24% of extractable compounds.

### 3.2. Effect on Vessels

In the present study, we evaluated the vasogenic potential and safety of application of extract from *Phellodendron amurense* Rupr. in the in ovo model (Table 2). This model is based on the observation of changes in the vessel of the chorioallantoic membrane of fertilized eggs.

The control (saline solution) and test solutions of PA with a concentration of 0.1 mg/mL did not cause a detectable effect after 5 min. On the other side, the administration of the PA extract with a concentration of 1.0 mg/mL may result in a slight irritant effect on vessels after 5 min of the application with the risk of hyperaemia. The extract in a concentration of 10.0 mg/mL caused hyperaemia (after 2 min) and haemorrhage (after 5 min), which is considered moderate irritation. Throughout the examination, none of the tested concentration caused coagulation of the CAM vasculature. Our results show that the increasing concentration of the PA causes the irritation of the CAM blood vessels.

### 3.3. Antiproliferative Activity

**Figure 5:**
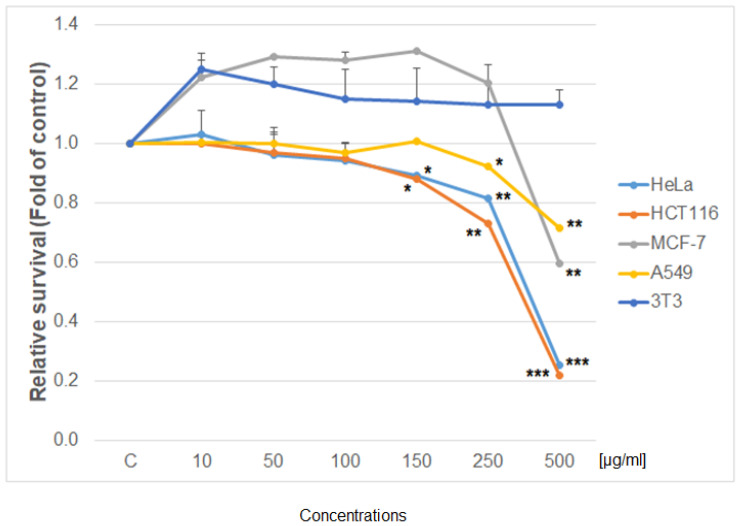
Relative survival of tested cell lines treated with *Phellodendron* extract (10–500 µg/mL) and analysed by MTS metabolic assay. Data were obtained from three independent experiments, and significant differences were marked as *p* < 0.05 (*), *p* < 0.01 (**), *p* < 0.001 (***) versus control cells (untreated cells).

**Figure 6:**
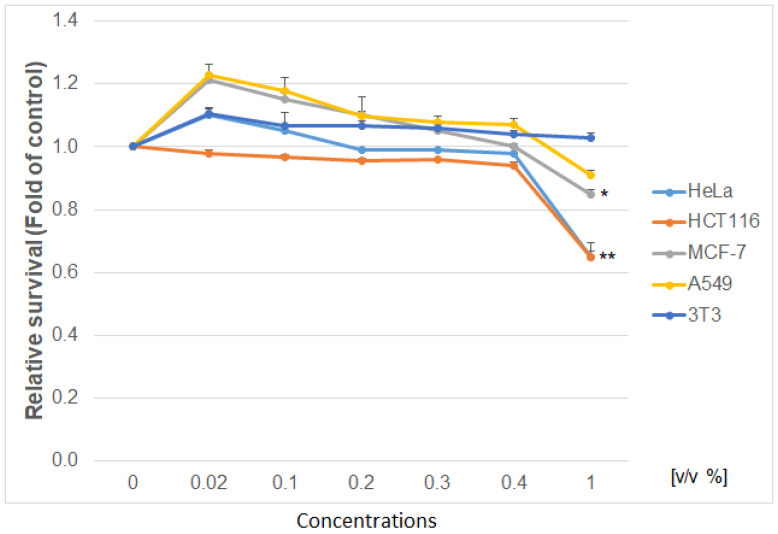
Relative survival of tested cell lines treated with vehicle (60% ethanol) (0.02–1 *v*/*v* %) and analysed by MTS metabolic assay. Data were obtained from three independent experiments, and significant differences were marked as *p* < 0.05 (*), *p* < 0.01 (**) versus control cells (untreated cells).

**Figure 7:**
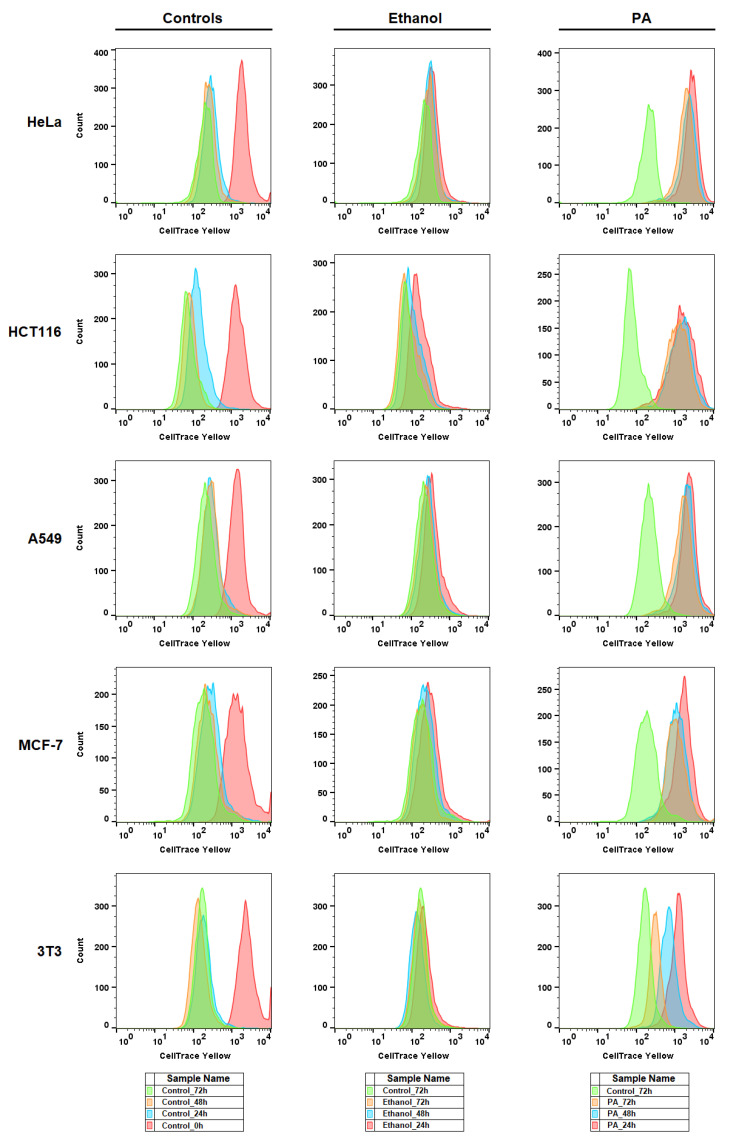
Flow cytometry analysis of tested cells proliferation stained with CellTrace^TM^ Yellow in controls group, ethanol vehicle and PA tincture (IC50)-treated cells. Representative histograms of two independent experiments are presented.

Using the colorimetric, metabolic MTS and Flow cytometry CellTrace^TM^ Yellow assay, the antiproliferative effect of the studied extract was determined as shown in Figure 5, Figure 6 and Figure 7. The IC50 values of the PA tincture on human cancer (HeLa—cervical carcinoma cells; MCF-7—breast cancer cells; HCT 116—human colorectal carcinoma; A549—lung carcinoma) and non-cancer (3T3—murine fibroblasts) cell lines calculated by MTS test are presented in Table 3. When comparing the metabolic activity of PA on different cancer cell lines (Figure 5), the strongest effect was shown on cell proliferation of HCT 116 and HeLa cells followed by MCF-7 and A549 cells. Moreover, the survival of non-cancerous murine fibroblast 3T3 exposed to PA tincture was not affected in the tested concentration range. Vehiculum used for the preparation of the PA tincture (60% ethanol) has no effect on all tested cells (except the highest *v*/*v* 1%) compared to untreated cells (Figure 6). Furthermore, the flow cytometry proliferation test (Figure 7) of the PA tincture IC50 values for every tested cancer cell line showed strong proliferation inhibition in a time-dependent manner (24–72 h) compared to untreated cells and also to vehicle-treated cells. The 3T3 fibroblast after higher IC50 value from A549 cells showed only partial inhibition of proliferation with minimal loss in viability. These findings indicated a high selectivity and specific cell-type-dependent toxicity of PA bark tincture.

### 3.4. Cell Cycle Analysis

Cell cycle distribution and changes were evaluated after tincture of PA (IC50) treatment in all cancer cell lines and 3T3 fibroblasts. As the result showed (Table 4, Table 5, Table 6, Table 7 and Table 8), PA treatment caused time-dependent G1 phase cell cycle arrest in HeLa (24 h), HCT116 (24 h) and A549 (24–48 h) cells. MCF-7 cells showed no cell cycle block. Moreover, PA treatment led to an increase in the sub-G0 population in a time-dependent manner in all cancer cell lines except A549 due the long persistence of the G1 block. The sub-G0 population is evaluated as apoptotic with fragmented DNA. With increased sub-G0 population and G1 arrest, the G1 population consequently decreased, mainly at 72 h after treatment. On the other hand, ethanol treatment as PA solvent caused no significant cell cycle changes in all tested cell lines. Furthermore, the treatment of the 3T3 fibroblast with the tincture of PA bark or 60% ethanol showed no significant changes.

### 3.5. Artemia Franciscana Test

Screening test of cytotoxicity of PA bark was performed using *A. franciscana* (Table 9). We did not observe signs of toxicity after 24 and also after 48 h. We noted 16 ± 0.5% lethality after 72 h. After 96 hours, lethality increased to 34 ± 0.82% for the *Phellodendron amurense* extract. The lethality, which began to appear up to 72 h, shows the negative effect of *Phellodenron amurense* on *A. franciscana*.

### 3.6. Antiangiogenic Activity

The CAM assay was used to examine whether *Phellodendron amurense* has any effect on neovascularization in ovo. The inhibition of angiogenesis was analysed by two methods, which gave us information about total branching points and total vascular zone, which means the vessels’ density.

**Figure 8:**
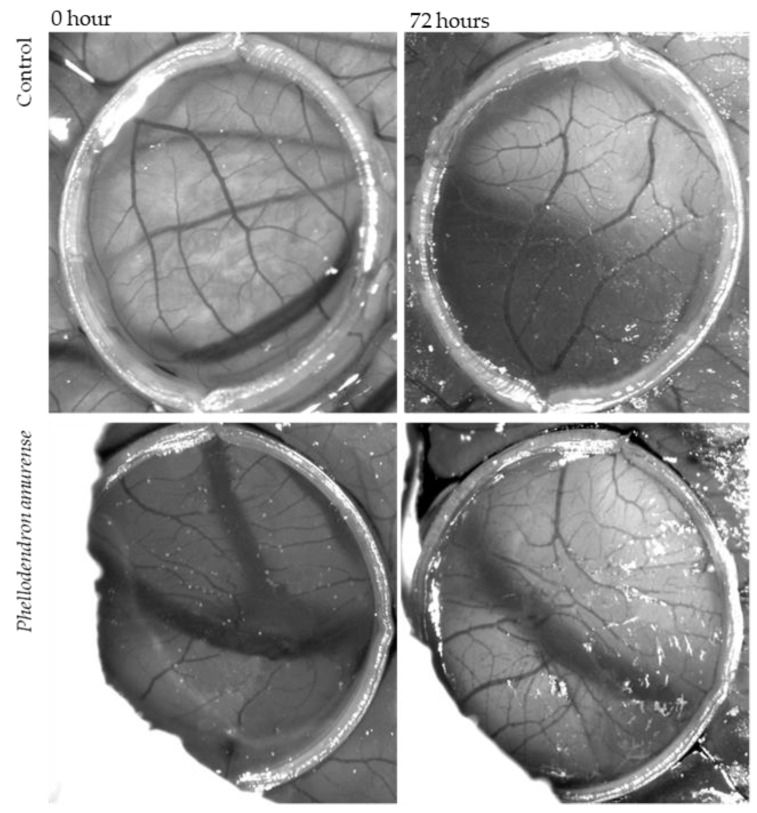
Photographs of HET-CAM without sample at the beginning of the experiment and after 72 h. HET-CAM treated by *Phellodendron amurense* Rupr. bark solution at the beginning of experiment and after 72 h.

**Figure 9:**
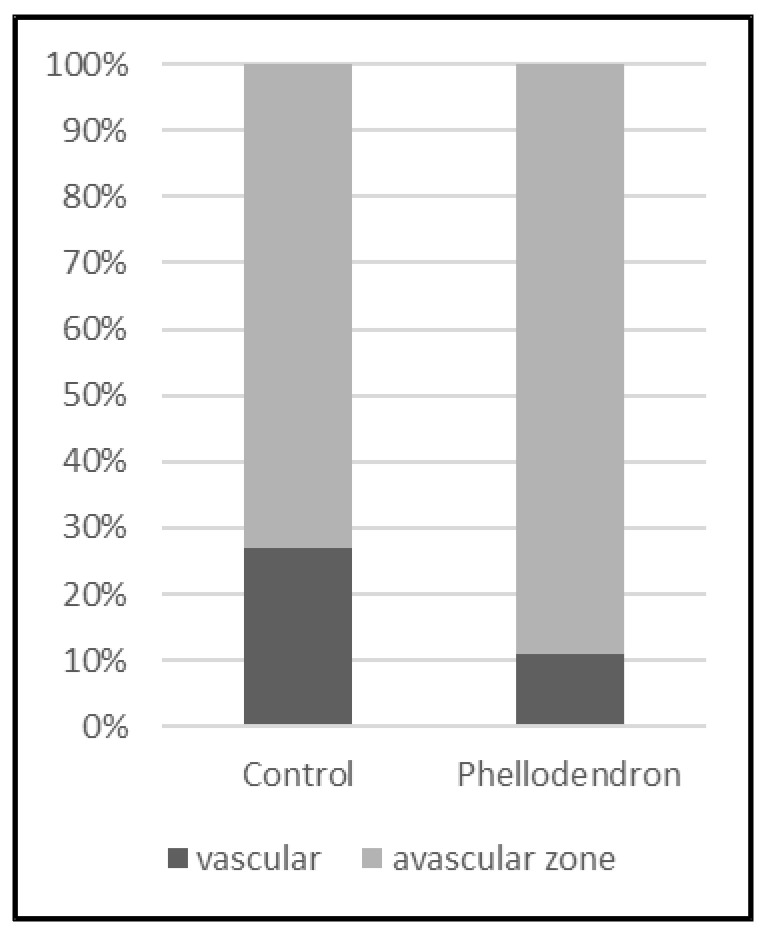
Graf of vascular and avascular zone of CAM without sample (control, (**left**)) and with *Phellodendron amurense* Rupr. bark solution (**right**).

The anti-angiogenic effect of dry PA residues from tincture dissolved in saline solution (1 mg/mL) on the treated CAM is shown in Figure 8. It was microscopically observed that tested solutions of PA inhibited angiogenesis in the CAM, causing the reduction in the vascular density compared to the control (saline solution) (Figure 9). The results can also be interpreted in the percentage of vessels density. Whereas in the case of control 27.5% of the figure can be considered vessels, this value is only 10.9% for the CAM treated with tested solutions of PA bark.

The second method for measuring the antiangiogenic potential of the tested PA solution was based on the enumeration of bifurcation points. An increase in the number of vessels crossing the delimited area was observed in the control (saline solution) (2.4 ± 0.1) and in CAM exposed to PA (1.7 ± 0.4). The present result demonstrated that *Phellodendron amurense* Rupr. exhibited a significant decrease in the branching of new capillaries from the existing basal vessels compared to the control after 72 h of treatment.

## 4. Discussion

Although evidence from epidemiological and animal studies suggests that increased consumption of a plant-based diet can reduce the risk of cancer, presently, little information is available concerning the use of botanicals in antiangiogenetic therapy. As a step toward developing botanicals as antiangiogenic agents, we examined the effect of *Phellodendron* on angiogenesis and on cancer cells.

Polyphenols were presented in prepared extract only in small concentrations. On the other hand, the main component of PA is berberine, which demonstrated different activity and antitumor properties [19,35]. The highest concentration of isoquinoline alkaloids was detected by spectrophotometer in *Tinctura* (49.4 mg/L ± 0.75 mg/L). It means that ethanol is better for the extraction of berberine and other isoquinoline alkaloids from PA bark than water. UHPLC analysis detected berberine in the concentration of 32.96 mg/kg. In a previous study, the content of berberine was abundant in the Me OH extract of *P. amurense* bark compared to other compounds. Its percentage yield was detected as 1.4% [36].

At first, the low toxicity was verified on vessels of PA by in ovo irritancy test. The present data show no significant signs of the irritancy, such as vasoconstriction, hyperaemia, haemorrhage or coagulation for the concentrations of 0.10 and 1.0 mg/mL of the berberine extract, which indicates its harmless character and the possibility of potential intravenous application. Moderate irritancy was observed only in the highest applied concentration 10 mg/mL. Although we have not recorded vascular irritancy, we evaluated the PA plant as safe and suitable for the production of potential new drugs, which can also be used by intravenous administration. The result of safety application of PA correlate with the observed minimal negative effects of orally administered product with PA in human test subjects [2,10,18]. Only few subjects described gentle stomach discomfort such as nausea [19]. The safety of application of our tested extract was detected in all tested concentrations; however, with growing concentration, the irritation was growing. A similar trend was observed in the case of administering snake venom [37]. No symptoms of haemorrhage, coagulation or mortality in embryo were observed after exposure of CAM to extract from lichen *Pseudevernia furfuracea* (L.) Zopf and its metabolite, physodic acid [38].

Recently, the bark of PA and its main component berberine have been demonstrated antitumor properties. We confirmed concentration and time-dependent cytotoxic and antiproliferative potential of PA in several cancer cell lines with different origin, mainly HCT116 (colon), HeLa (cervix), MCF-7 (breast) and A549 (lung). Our results are in accordance with several studies where, in the past decade, the number of reports demonstrating anti-cancer properties of commercial Asian origin Nexrutine^®^ in melanoma [17], prostate [39], pancreatic [40] and breast [6] in vitro models increased. Furthermore, we confirmed that proliferation inhibition in all tested cell lines except MCF-7 was caused by G1 cell cycle arrest and apoptosis, similar as described [6] in SkBr3 and MDA-MB-231 breast cancer model or [41] in A549 and H1299 lung carcinoma models. Berberine as the main component has stronger activity than PA. It was reported that the IC50 of berberine in HeLa cell was around 0.3 mM after 48 h [42] (B. Lu et al./Toxicology in Vitro 24 (2010) 1482–1490) and 0.14 mM in A549 after 24 h of incubation Oncol Lett June 2012; 3(6): 1263–1267) [43]. Moreover, the selectivity index indicated high selectivity for every cancer cell and potential non-toxic effects on normal healthy cells. These finding suggest the great potential of PA in clinical studies where PA could be well-tolerated after treatment of patients. This suggestion confirmed study of Swanson et al., where Nexrutine^®^ tolerance by prostate cancer patients was high, with no grade 4 toxicity [18]. For the combined Nexrutine^®^ and radiation component, no additional patients suffered from grade 3 toxicity.

The present study showed the anti-angiogenic activity tested in ovo of *Phellodendron amurense* Rupr. for the first time. Both methods of measuring anti-angiogenic effects were compared and showed very similar results. In both cases (vascular zone and vessel sprouting), a reduction in angiogenesis was found. *Phellodendron amurense* Rupr. was able to significantly induce blood vessel response to stimuli. These results suggested that PA could be particularly attractive for the treatment of cancer according to its antiangiogenic properties. Moreover, the extract of *Phellodendron amurense* Rupr. did not affect the embryo viability.

To the best of our knowledge, there is no information about the anti-angiogenic efficiency of ethanolic tincture of *Phellodendron amurense* Rupr. in vitro or in vivo. The anti-angiogenic effect of hot water extract was observed in vitro HUVECs and ex vivo in the rat aorta [44]. The anti-angiogenic effect was also tested in combination with other extracts in traditional Chinese herbal medicine, Qing-Luo-Yin (*Sophora flavescens* Ait., *Phellodendron amurense* Rupr., *Sinomenium acutum* Rehd. et Wils. and *Dioscorea hypoglauca* Palib) for the treatment of rheumatoid arthritis [45]. The main alkaloid in *Phellodendron amurense* Rupr. bark is the isoquinoline alkaloid, berberine. The influence of berberine on angiogenesis was tested by different models, but no CAM analysis was performed in ovo. In vivo, berberine showed antiangiogenic activity on B16F-10 melanoma cells and in C57BL/6 mice [46]. The main alkaloid from *Phellodendron* is berberine, which prevents non-alcoholic steatohepatitis-derived hepatocellular carcinoma by inhibiting inflammation and angiogenesis in mice [47]. Berberine could reduce metastasis and angiogenesis of cervical cancer cells, so it can be applied as a complementary treatment in metastasis control [48]. On the other hand, berberine also promotes angiogenesis. It was observed in an animal model that berberine promotes ischemia-induced angiogenesis in mice heart [49].

## 5. Conclusions

In summary, it appears from the results of this in vitro, in vivo and in ovo pilot study that *Phellodendron amurense* bark extract has a good anti-proliferative effect based on its anti-angiogenic effect. All mentioned effects were connected with minimal negative effect, as shown by the HET-CAM irritation test. A significant reduction in vessel sprouting and a higher percentage of avascular zone was observed after the application of the extract, which is a proof of its anti-angiogenic potential. The anti-proliferative effect was determined in vitro on human lung, breast, colorectal as well as cervical carcinoma cells and also in vivo on *Artemia franciscana*. All used tests are cost-effective, animal- and human-friendly alternative models which reduce the number of experimental animals. The obtained results could contribute to the development of novel drugs based on the extracts from *Phellodendron amurense* for the treatment of cancer or other diseases, in which angiogenesis plays a significant role.

## Figures and Tables

**Table 1:** Concentration of polyphenols and isoquinoline alkaloids in different extracts of *Phellodendron amurense* Rupr. bark.

Metabolites	Polyphenols [g/L]	Isoquinoline Alkaloids [mg/L]
*Infusum*	1.61 ± 0.039	2.74 ± 0.05
*Infusum frigidum*	1.37 ± 0.033	24.1 ± 0.04
*Decoctum*	0.70 ± 0.017	8.43 ± 0.02
*Tinctura*	0.50 ± 0.012	49.4 ± 0.75

**Table 2:** Vasoactivity of *Phellodendron amurense* Rupr. bark.

Vasoactivity	Hyperaemia			Haemorrhage			Coagulation			Cumulative Score	Irritation Assessment
Time [min]	0.5	2	5	0.5	2	5	0.5	2	5		
saline sol.										0	Negligible
0.1 mg/mL										0	Negligible
1 mg/mL			1							1	Slight
10 mg/mL		3				3				6	Moderate

**Table 3:** IC50 values of extract and the ones calculated to concentration of berberine and Selectivity index calculated from MTS metabolic assay results.

	Cancer Cell Lines				Fibroblasts
	HeLa	HCT116	MCF-7	A549	3T3
IC 50 extract [µg/mL]	377.5 ± 8.1	356.7 ± 3.0	539.5 ± 4.5	753.6 ± 0.8	3448.7 ± 201.6
IC 50 berberine [µM]	76.5 ± 6.9	69. 3 ± 3.6	70.9 ± 4.1	195.1 ± 15.8	247.5 ± 13.8
IC 50 berberine [µg/mL] (calculated)	0.0124	0.0117	0.0177	0.0248	0.1136
IC 50 berberine [µM] (calculated)	36.98	34.96	52.86	73.85	337.94
Selectivity index extract	9.1	9.6	6.4	4.6	1
Selectivity index berberine	3.2	3.6	3.5	1.3	1

**Table 4:** Cell cycle analysis of HeLa cells after 24, 48 and 72 h incubation with PA tincture (IC50).

HeLa	sub-G0	G1	S	G2/M
Control 24 h	1.5 ± 0.06	70.7 ± 1.06	11.1 ± 0.45	16.8 ± 0.53
Ethanol	1.6 ± 0.07	70.6 ± 1.47	10.1 ± 0.38	17.7 ± 1.14
PA tincture	2.3 ± 0.47	80.8 ± 0.01 *^+^	5.2 ± 0.84 *^+^	11.7 ± 1.98 *^+^
Control 48 h	3.0 ± 0.24	77.1 ± 1.22	9.0 ± 0.06	10.8 ± 1.54
Ethanol	2.4 ± 0.26	77.6 ± 1.51	9.5 ± 0.27	10.5 ± 1.53
PA tincture	6.1 ± 0.03	75.3 ± 1.14	7.8 ± 1.01	10.8 ± 2.14
Control 72 h	1.6 ± 0.20	80.0 ± 0.33	7.9 ± 0.07	10.5 ± 0.61
Ethanol	2.9 ± 0.02	79.0 ± 1.02	7.9 ± 0.02	10.3 ± 1.03
PA tincture	7.6 ± 0.10 *^+^	66.0 ± 1.80 **^+^	12.8 ± 0.73	13.6 ± 0.98

Significance: *p* < 0.05 (*), *p* < 0.01 (**) vs. untreated control and *p* < 0.05 (+) vs. vehicle (ethanol)-treated cells.

**Table 5:** Cell cycle analysis of HCT116 cells after 24, 48 and 72 h incubation with PA tincture (IC50).

HCT116	sub-G0	G1	S	G2/M
Control 24 h	0.7 ± 0.03	40.9 ± 0.01	23.7 ± 1.80	34.7 ± 1.80
Ethanol	1.3 ± 0.28	37.2 ± 1.50	26.9 ± 1.27	34.6 ± 0.10
PA tincture	6.0 ± 0.01 *^+^	67.1 ± 2.70 **^++^	11.1 ± 1.74 **^+^	15.9 ± 1.63 **^++^
Control 48 h	0.5 ± 0.05	49.7 ± 1.28	22.4 ± 1.47	27.4 ± 2.40
Ethanol	0.6 ± 0.01	50.3 ± 0.15	23.0 ± 2.25	26.2 ± 1.78
PA tincture	6.2 ± 0.67 *^+^	55.6 ± 1.40 *^+^	22.0 ± 1.55	16.3 ± 1.10 *^+^
Control 72 h	1.7 ± 0.60	72.6 ± 3.10	10.1 ± 2.09	15.5 ± 1.45
Ethanol	1.8 ± 0.27	73.6 ± 3.68	10.5 ± 2.04	14.5 ± 2.41
PA tincture	9.9 ± 0.90 *^+^	59.1 ± 1.02 *^+^	14.0 ± 1.22	18.2 ± 1.67

Significance: *p* < 0.05 (*), *p* < 0.01 (**) vs. untreated control and *p* < 0.05 (+), *p* < 0.01 (++) vs. vehicle (ethanol)-treated cells.

**Table 6:** Cell cycle analysis of A549 cells after 24, 48 and 72 h incubation with PA tincture (IC50).

A549	sub-G0	G1	S	G2/M
Control 24 h	0.2 ± 0.12	66.1 ± 1.40	19.6 ± 2.63	14.1 ± 1.39
Ethanol	0.9 ± 0.22	69.5 ± 1.60	16.4 ± 2.40	13.2 ± 1.78
PA tincture	0.7 ± 0.38	83.3 ± 2.70 **^+^	8.6 ± 1.47 *^+^	7.4 ± 1.62 *^+^
Control 48 h	0.8 ± 0.09	70.0 ± 2.53	14.8 ± 2.25	14.5 ± 0.37
Ethanol	0.6 ± 0.15	71.6 ± 2.37	15.7 ± 1.88	12.1 ± 0.65
PA tincture	0.8 ± 0.33	79.4 ± 0.37 *^+^	10.1 ± 0.67 *^+^	9.7 ± 0.66 *^+^
Control 72 h	1.1 ± 0.24	75.7 ± 0.24	12.7 ± 0.61	10.5 ± 0.63
Ethanol	0.5 ± 0.06	75.5 ± 0.73	12.6 ± 0.65	11.4 ± 1.46
PA tincture	0.8 ± 0.20	78.6 ± 0.47	9.8 ± 2.65	10.8 ± 1.35

Significance: *p* < 0.05 (*), *p* < 0.01 (**) vs. untreated control and *p* < 0.05 (+) vs. vehicle (ethanol)-treated cells.

**Table 7:** Cell cycle analysis of MCF-7 cells after 24, 48 and 72 h incubation with PA tincture (IC50).

MCF-7	sub-G0	G1	S	G2/M
Control 24 h	1.6 ± 0.93	58.3 ± 1.22	15.4 ± 0.20	24.8 ± 2.37
Ethanol	1.6 ± 0.01	62.1 ± 1.02	15.9 ± 0.33	20.5 ± 1.35
PA tincture	7.1 ± 0.61 *^+^	59.8 ± 0.98	15.0 ± 1.31	18.1 ± 0.29 *
Control 48 h	1.3 ± 0.11	63.9 ± 0.08	13.4 ± 0.29	21.5 ± 0.08
Ethanol	2.1 ± 0.48	64.4 ± 0.07	11.4 ± 0.65	22.1 ± 1.06
PA tincture	9.9 ± 0.17 *^+^	60.7 ± 1.56	11.9 ± 0.52	17.5 ± 0.57 *^+^
Control 72 h	1.5 ± 0.22	69.4 ± 0.78	9.9 ± 0.04	19.2 ± 1.06
Ethanol	1.7 ± 0.44	73.6 ± 1.76	9.6 ± 0.02	15.2 ± 2.20
PA tincture	12.2 ± 0.94 *^+^	59.1 ± 0.90 *^+^	14.5 ± 1.89	14.2 ± 1.98 *

Significance: *p* < 0.05 (*) vs. untreated control and *p* < 0.05 (+) vs. vehicle (ethanol)-treated cells.

**Table 8:** Cell cycle analysis of 3T3 cells after 24, 48 and 72 h incubation with PA tincture (IC50).

3T3	sub-G0	G1	S	G2/M
Control 24 h	1.0 ± 0.26	63.0 ± 1.04	15.5 ± 1.51	20.5 ± 2.22
Ethanol	1.2 ± 0.56	60.4 ± 2.62	18.6 ± 1.57	19.9 ± 2.86
PA tincture	1.1 ± 0.58	65.5 ± 0.20	14.7 ± 1.54	18.8 ± 2.18
Control 48 h	1.0 ± 0.34	63.4 ± 1.29	17.9 ± 1.39	17.8 ± 1.55
Ethanol	0.7 ± 0.42	65.7 ± 1.60	17.7 ± 0.24	15.9 ± 1.79
PA tincture	1.1 ± 0.36	61.8 ± 1.18	18.8 ± 1.79	18.3 ± 1.56
Control 72 h	0.5 ± 0.04	68.9 ± 2.59	12.5 ± 0.69	18.2 ± 2.72
Ethanol	1.0 ± 0.14	64.9 ± 0.38	17.8 ± 2.92	16.3 ± 1.84
PA tincture	0.3 ± 0.25	65.7 ± 1.51	13.4 ± 1.46	20.6 ± 0.85

Significance: N/A.

**Table 9:** Lethality [%] of nauplii *Artemia franciscana* after application of *Phellodendron amurense* Rupr. extract.

Time	24 h	48 h	72 h	96 h
Control	0%	0%	4 ± 0.58%	8 ± 0.5%
Extract	0%	0%	16 ± 0.5% *	34 ± 0.82% *

Significance: *p* < 0.05 (*) vs. untreated control.

## Data Availability

Not applicable.
